# Risk factors for mortality in critically ill patients with COVID-19: a multicenter retrospective case-control study

**DOI:** 10.1186/s12879-021-06300-7

**Published:** 2021-06-24

**Authors:** Jinghua Gao, Li Zhong, Ming Wu, Jingjing Ji, Zheying Liu, Conglin Wang, Qifeng Xie, Zhifeng Liu

**Affiliations:** 1 The First School of Clinical Medicine, Southern Medical University, Guangzhou, 510010 China; 2 Department of Critical Care Medicine, General Hospital of Southern Theater Command of PLA, Guangzhou, 510010 China; 3 Department of Critical Care Medicine, The First Affiliated Hospital, Guizhou University of Chinese Medicine, Guiyang, 550001 China; 4 Department of Critical Care Medicine and Infection Prevention and Control, Health Science Center, The Second People’s Hospital of Shenzhen & First Affiliated Hospital of Shenzhen University, Shenzhen, 518035 China; 5 Key Laboratory of Hot Zone Trauma Care and Tissue Repair of PLA, General Hospital of Southern Theater Command of PLA, Guangzhou, 510010 China

**Keywords:** SARS-COV-2, COVID-19, Risk factors, Mortality

## Abstract

**Background:**

Coronavirus disease 2019 (COVID-19) has spread around the world, until now, the number of positive and death cases is still increasing. Therefore, it remains important to identify risk factors for death in critically patients.

**Methods:**

We collected demographic and clinical data on all severe inpatients with COVID-19. We used univariable and multivariable Cox regression methods to determine the independent risk factors related to likelihood of 28-day and 60-day survival, performing survival curve analysis.

**Results:**

Of 325 patients enrolled in the study, Multi-factor Cox analysis showed increasing odds of in-hospital death associated with basic illness (hazard ratio [HR] 6.455, 95% Confidence Interval [CI] 1.658–25.139, *P* = 0.007), lymphopenia (HR 0.373, 95% CI 0.148–0.944, *P* = 0.037), higher Sequential Organ Failure Assessment (SOFA) score on admission (HR 1.171, 95% CI 1.013–1.354, *P* = 0.033) and being critically ill (HR 0.191, 95% CI 0.053–0.687, *P* = 0.011). Increasing 28-day and 60-day mortality, declining survival time and more serious inflammation and organ failure were associated with lymphocyte count < 0.8 × 109/L, SOFA score > 3, Acute Physiology and Chronic Health Evaluation II (APACHE II) score > 7, PaO2/FiO2 < 200 mmHg, IL-6 > 120 pg/ml, and CRP > 52 mg/L.

**Conclusions:**

Being critically ill and lymphocyte count, SOFA score, APACHE II score, PaO2/FiO2, IL-6, and CRP on admission were associated with poor prognosis in COVID-19 patients.

**Supplementary Information:**

The online version contains supplementary material available at 10.1186/s12879-021-06300-7.

## Introduction

Coronavirus disease 2019 (COVID-19) is a serious infectious disease caused by infection with severe acute respiratory syndrome coronavirus 2 (SARS-COV-2, previously known as 2019-nCoV) [1], and has caused widespread infection in just a few months. Worldometer (world real-time statistics) showed that, as of March 12, 2021, the cumulative number of confirmed cases of COVID-19 in the world exceeded 119 million, and cumulative deaths exceeded 2.6 million. According to the latest report, the mortality rate of COVID-19 was 5.6–20.3% [2, 3], while the mortality rate in severe patients can reach 30–60% [4–6]. An early and accurate evaluation of the severity of inpatients is very important, as it may improve the prognosis and treatment. A recent Meta-analysis have reported that the risk factors for higher mortality from COVID-19 include older age, male gender, diabetes, and hypertension [3, 7], but there are few cases with supporting evidence, especially for severe COVID-19 patients with a high risk of death, which can provide a reference for future treatment [8, 9].

## Methods

### Study design and participants

This multicenter retrospective case-control study was performed in four government-designated treatment units for COVID-19 patients from 3 cities of China. All patients who were diagnosed with severe COVID-19 (defined as Severe and Critical according to Chinese Guidelines) were screened, and those who died or were discharged between January, 2020 and March, 2020 met the inclusion criteria.

The study was approved by the Research Ethics Commission of the General Hospital of Southern Theater Command of PLA (HE-2020-08) and the requirement for informed consent was waived by the Ethics Commission.

Inclusion Criteria: (1) Adult aged > 18 years; (2) Laboratory (reverse transcription polymerase chain reaction) confirmed SARS-COV-2 infection in throat swab and/or sputum and/or lower respiratory tract samples; or conformed plasma positive of specific antibody (immunoglobulin [Ig] M IgM or and IgG) against SARS-COV-2; (3) In-hospital treatment for ≥72 h; and (4) Meeting any of the following criteria for severe (a-c) or critical (d-f) diagnosis: (a) respiratory rate > 30/min; (b) resting oxygen saturation < 90%; or (c) PaO2/FiO2 ratio < 300 mmHg; (d) respiratory failure and needing mechanical ventilation; (e) shock occurs; or (f) multiple organ failure and needing Intensive Care Unit (ICU) monitoring.

Exclusion Criteria: 1) Existence of other explanations for pneumonia, including but not limited to: influenza A virus, influenza B virus, bacterial pneumonia, fungal pneumonia, or noninfectious causes; (2) women who are pregnant or breast-feeding.

### Research procedures

Basic characteristics of patients were collected, which consisted of demographic information; comorbidities; diagnostic classification; and indicators of inflammation and organ function of patients on admission, including complete blood count, procalcitonin (PCT), C-reactive protein (CRP), interleukin-6 (IL-6), liver function, kidney function, blood lactic acid concentration, oxygenation index, Acute Physiology and Chronic Health Evaluation II (APACHE II) score and Sequential Organ Failure Assessment (SOFA) score. We divided all patients into the survival group and the death group according to their 60-day survival, and compared the characteristics of groups on admission. Univariable and multivariable Cox regression analyses were used to explore the independent risk factors for death in severe inpatients with COVID-19. These indicators, encompassing lymphocyte count, SOFA score, APACHE II score, Pa02/FiO2, IL-6, CRP, and clinical classification, were used for subgroup analysis and survival curve analysis.

### Definitions

“Critical COVID-19” in this article is defined as a term combining patients with “Severe” and “Critical” COVID-19, classified following Chinese Recommendations for Diagnosis and Treatment of Novel Coronavirus (SARS-COV-2) infection (Trial 7th version) published by the National Health Commission of China [10]. Sepsis was defined according to the 2016 Third International Consensus definition for sepsis and septic shock [11].

### Statistical analysis

The categorical data were summarized as numbers and percentages, and inter-group comparisons were performed using Mann-Whitney U test, χ2 test or Fisher’s exact test. Continuous variables were expressed as the median and interquartile range (IQR). Continuous data with Gaussian distribution were compared with the Student’s t test or one-way ANOVA; those with a non-Gaussian distribution were compared with the Wilcoxon rank-sum test. The patient endpoint event was death within 60 days after disease onset. The survival curve was drawn using the Kaplan-Meier method. Significant indicators were analyzed using single factor analysis; *P* < 0.1 indicators were included in the multifactor Cox regression model, and forward LR was used to gradually eliminate them. The impact of each indicator on prognosis was analyzed, and prognostic risk factors were screened. Statistical analysis was performed using the SPSS Windows version 11.0 statistical package (SPSS Inc., Chicago, IL) and *P* values (two-tailed) below 0.05 were considered statistically significant.

## Results

### Demographics and baseline characteristics

**Fig. 1:**
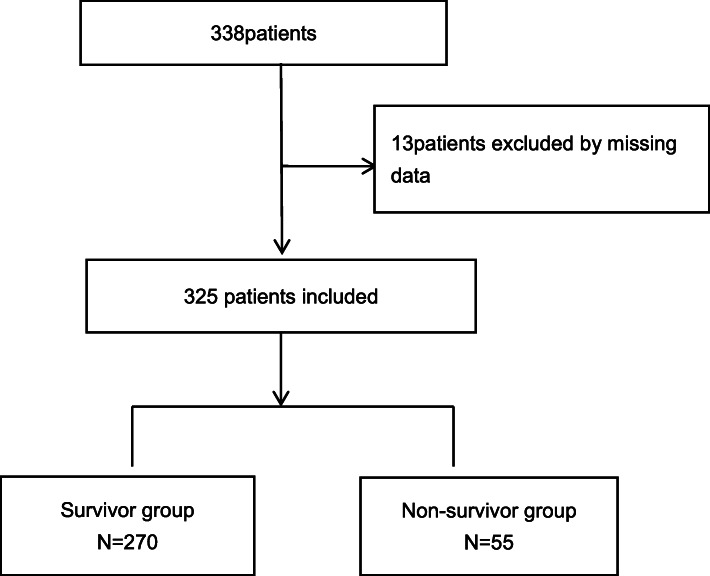
Flow chart of all excluded and included patients

A total of 338 patients who fulfilled the admission criteria of this study were collected before March 2020. After excluding 13 patients without available clinical data, we included 325 patients (Fig. 1) in the final analysis, of whom 270 (83%) survived and 55 (17%) died. According to the severity of the disease, the patients were divided into 222 cases of severe (68%) and 103 cases of critical (32%) patients, and 6 cases of severe (11%) and 49 cases of critical (89%) non-survivors. Significant differences in age, underlying diseases, and clinical classification were found between survivors and non-survivors (*p* < 0.05), suggesting that older age, comorbidity, and greater disease severity were associated with a higher mortality rate (Table 1).

**Table 1:** Baseline characteristics of demographics, clinical and laboratory findings in Survivor group and Non-Survivor group

	Total (***N*** = 325)	Survivor (***N*** = 270)	Non-Survivor (***N*** = 55)	***P*** value
**Demographics,clinical characteristics**				
Age, years	58.0 (46.0–69.0)	56.0 (42.0–65.0)	70.0 (62.0–82.0)	< 0.00
Sex N (%)				1
Male	189 (58%)	154 (57%)	35 (64%)	0.366
Female	136 (42%)	116 (43%)	20 (37%)	
Comorbidity N(%)	155 (48%)	117 (43%)	38 (69%)	0.001
Hypertension	98 (30%)	71 (26%)	27 (49%)	0.001
Coronary heart disease	31 (10%)	20 (7%)	11 (20%)	0.004
Chronic kidney disease	5 (2%)	2 (1%)	3 (6%)	0.036
Diabetes	38 (12%)	26 (10%)	12 (22%)	0.010
Chronic obstructive lung	10 (3%)	6 (2%)	4 (7%)	0.070
Stroke	16 (5%)	7 (3%)	9 (16%)	< 0.001
Carcinoma	10 (3%)	8 (3%)	2 (4%)	0.679
Other	61 (19%)	45 (17%)	16 (29%)	0.030
Temperature(°C),median(IQR)	37.0 (36.5–37.8)	37.0 (36.6,37.9)	36.6 (36.3–37.4)	0.014
Pulse(beats per min), median(IQR)	88 (80.0–97.0)	87.0 (80.0,96.0)	90.0 (80.3–99.5)	0.356
Respiratory rate(breaths per min), median(IQR)	20.0 (20.0–23.0)	20.0 (19.0,22.0)	23.0 (20.0–26.0)	< 0.001
Systolic blood pressure, median(IQR)	127.0 (117.0–138.0)	125.0 (115.0,138.0)	130.5 (127.3–146.5)	0.003
Diastolic blood pressure, median(IQR)	78.0 (70.0–85.0)	78.0 (70.0,85.0)	76.0 (65.8–85.0)	0.222
APACH II sore, median(IQR)	6.0 (4.0–9.0)	5.0 (3.0–7.0)	13.0 (9.0–29.0)	< 0.001
SOFA sore, median(IQR)	2.0 (2.0–4.0)	2.0 (1.0,3.0)	7.0 (4.0–14.0)	< 0.001
Clinical Classifications N (%)				< 0.001
Severe type	222 (68%)	216 (80%)	6 (11%)	
Critical type	103 (32%)	54 (20%)	49 (89%)	
In-hospital days	20.0 (14.0–28.0)	20.0 (14.0–28.0)	15.0 (7.0–28.0)	0.003
Total course of disease ^a^	28.0 (19.0–37.0)	27.0 (19.0–36.0)	30.0 (20.0–38.0)	0.345
**Laboratory findings, median(IQR)**				
WBC, (1 × 109/L)	5.8 (4.2–8.3)	5.3 (4.1–7.1)	9.2 (5.6–15.7)	< 0.001
NEU,(1 × 109/L)	3.9 (2.6–6.5)	3.6 (2.4–5.4)	8.1 (4.8–14.4)	< 0.001
LYM,(1 × 109/L)	1.0 (0.6–1.4)	1.0 (0.7–1.5)	0.6 (0.4–0.8)	< 0.001
MON,(1 × 109/L)	0.4 (0.3–0.6)	0.4 (0.3–0.6)	0.4 (0.2–0.6)	0.205
PLT,(1 × 109/L)	178.0 (144.0–233.5)	186.0 (147.5–239.5)	153.0 (84.5–199.5)	< 0.001
HGB,(g/L)	129.0 (117.0–141.0)	130.0 (119.5–142.5)	117.0 (94.0–133.5)	< 0.001
FIB,(g/L)	4.1 (3.4–4.8)	4.1 (3.4–4.8)	3.9 (3.0–4.6)	0.340
IL-6,(pg/ml)	19.1 (7.7–42.8)	16.0 (7.1–33.8)	66.9 (22.6–112.5)	< 0.001
PCT,(ng/ml)	0.1 (0–0.2)	0.1 (0–0.1)	0.2 (0.1–0.4)	< 0.001
CRP,(mg/L)	25.2 (8.7–63.6)	20.1 (7.9–46.3)	70.0 (34.9–150.5)	< 0.001
ALT, (U/L)	24.0 (16.1–37.9)	24.0 (16.0–37.4)	25.8 (18.0–43.8)	0.378
TBIL, (umol/L)	11.3 (7.9–15.6)	10.9 (7.5–15.0)	13.3 (10.2–20.1)	0.004
DBIL, (umol/L)	3.7 (2.4–6.1)	3.5 (2.2–5.2)	5.8 (3.6–8.6)	< 0.001
CREA, (μmol/L)	65.0 (52.5–80.9)	64.0 (52.0–80.0)	68.4 (55.3–104.8)	0.096
Lac, (mmol/L)	1.6 (1.2–2.2)	1.5 (1.1–2.1)	1.9 (1.3–2.5)	0.077
Pa0_2_/FiO_2_	237.9 (164.2–285.0)	247.3 (196.6–286.5)	143.3 (90.2–235.6)	< 0.001

^a^ Total course of disease:Time from illness onset to death or discharge, days; *IQR* Inter-Quartile Range, *APACHE II* Acute Physiology and Chronic Health Evaluation II score, *SOFA* Sequential Organ Failure Assessment, *WBC* White blood cell count, *NEU* Neutrophil, *LYM* Lymphocyte count, *MON* Monocytes, *PLT* Platelet count, *HGB* Hemoglobin, *FIB* Fibrinogen, *IL-6* Interleutin-6, *PCT* Procalcitonin, *CRP* C-reactive protein, *ALT* Alanine aminotransferase, *TBIL* Total bilirubin, *DBIL* Direct bilirubin, *CREA* Creatine, *Lac* lactic acid

The median time from admission to discharge was 20.0 days (IQR 14.0–28.0), whereas the median time from illness onset to death was 28.0 days (IQR 19.0–37.0). The median total hospitalization time of non-survivors was 15.0 days (IQR 7.0–28.0) and the median time of the total course of the disease was 30.0 days (20.0–38.0) (Table 1).

The physiological or pathological indexes of enrolled patients are shown in Table 1. Body temperature was 37.0 °C (IQR 36.5–37.8), heart rate was 88.0/min (IQR 80.0–97.0), respiratory rate was 20.0/min (IQR 20.0–23.0), blood pressure was 127.0 (IQR 117.0–138.0) /78.0 (70.0–85.0) mmHg, APACHE II score was 6.0 (IQR 4.0–9.0), SOFA score was 2.0 (IQR 2.0–4.0). Inflammation and organ function indexes of non-survivors were higher than those of the survivors (Table 1).

### Risk factors analysis for 60 day in-hospital mortality

We chose age, gender, comorbidity, SOFA score, APACHE II score, lymphocyte count, PCT, creatine, Pa02/FiO2, lactic acid, and clinical classification as variables for our multivariable Cox regression model. Results showed that comorbidity, critical state, reduced lymphocyte levels, and higher SOFA score are independent risk factors for increased 60-day mortality in patients (Table 2).

**Table 2:** Multivarate analysis for factors associated with death in hospital

Variable	Univariable HR (95%Cl)	*P* value	Multivarate HR(95%CI)	*P* value
Gender	1.290 (0.745–2.235)	0.363	0.660 (0.283–1.536)	0.335
Age	1.057 (1.038–1.076)	< 0.001	1.026 (0.987–1.067)	0.195
Comorbidity	2.635 (1.487–4.669)	0.001	6.455 (1.658–25.139)	0.007
APACHEII score	1.143 (1.117–1.170)	< 0.001	1.007 (0.923–1.098)	0.881
SOFA score	1.186 (1.152–1.222)	< 0.001	1.171 (1.013–1.354)	0.033
Lymphocyte	0.154 (0.071–0.331)	< 0.001	0.373 (0.148–0.944)	0.037
Procalcitonin	1.054 (1.017–1.093)	0.004	0.997 (0.953–1.044)	0.912
Creatine	1.009 (1.006–1.012)	< 0.001	1.005 (0.999–1.011)	0.080
Pa0_2_/FiO_2_	0.991 (0.987–0.995)	< 0.001	0.998 (0.994–1.003)	0.428
lactic acid	1.109 (0.968–1.270)	0.832	0.978 (0.624–1.534)	0.923
Clinical classification	0.044 (0.019–0.103)	< 0.001	0.191 (0.053–0.687)	0.011

*APACHE II* Acute Physiology and Chronic Health Evaluation II score, *SOFA* Sequential Organ Failure Assessment

### Subgroup analysis

Cutoff value obtained by curve fitting, including lymphocyte count (> 0.8 × 109/L and < 0.8 × 109/L) SOFA score (> 3 and < 3), APACHE II score (> 7 and < 7), PaO2/FiO2 (> 200 and < 200 mmHg), IL-6 (> 120 and < 120 pg/ml), CRP (> 52 and < 52), and clinical classification were processed for further subgroup analysis and survival curve analysis.

We found that lymphocyte count < 0.8 × 109/L, SOFA score > 3, APACHE II score > 7, PaO2/FiO2 < 200 mmHg, IL-6 > 120 pg/ml, and CRP > 52 mg/L were associated with greater 28-day and 60-day mortality in critical patients (*P* < 0.05) (Table 3), who have a more severe gingival inflammatory index and organ function index (*P* < 0.05) (Supplementary Tables 1, 2, 3, 4, 5, 6, 7). Survival curve analysis indicated patients with lymphocyte count > 0.8 × 109/L, SOFA score < 3, APACHE II score < 7, PaO2/FiO2 > 200 mmHg, IL-6 < 120 pg/ml, and CRP < 52 mg/L had a higher in-hospital survival rate (Supplementary Figures 1, 2, 3, 4, 5, 6, 7).

**Table 3:** The primary outcomes for all patients in different subgroups

Primary outcomes N(%)	Total (*N* = 304)	LYM>0.8 (*N* = 191)	LYM ≤ 0.8 (*N* = 113)	*P* value
28-day mortality	37 (12%)	10 (5%)	27 (24%)	**< 0.001**
60-day mortality	49 (16%)	13 (7%)	36 (32%)	< 0.001
	Total (*N* = 311)	SOFA>3.0 (*N* = 78)	SOFA≤3.0 ((*N* = 233)	*P* value
28-day mortality	36 (11%)	32 (41%)	4 (2%)	< 0.001
60-day mortality	47 (15%)	41 (53%)	6 (3%)	< 0.001
	Total (*N* = 302)	APACHE II>7.0 (*N* = 102)	APACHEII≤7.0 (*N* = 200)	*P* value
28-day mortality	36 (12%)	32 (31%)	4 (2%)	< 0.001
60-day mortality	47 (16%)	42 (41%)	5 (3%)	< 0.001
	Total (*N* = 215)	Pa0_2_/FiO_2_>200 (*N* = 140)	Pa0_2_/FiO_2_ ≤ 200 (*N* = 75)	*P* value
28-day mortality	31 (14%)	11 (8%)	20 (27%)	< 0.001
60-day mortality	42 (20%)	13 (9%)	29 (39%)	< 0.001
	Total (*N* = 186)	IL-6>120 (*N* = 11)	IL-6 ≤ 120 (*N* = 175)	*P* value
28-day mortality	20 (11%)	4 (36%)	16 (9%)	0.020
60-day mortality	27 (15%)	6 (55%)	21 (12%)	0.002
	Total (*N* = 296)	CRP>52 (*N* = 81)	CRP ≤ 52 (*N* = 215)	*P* value
28-day mortality	34 (12%)	20 (25%)	14 (7%)	< 0.001
60-day mortality	46 (16%)	28 (35%)	18 (8%)	< 0.001
	Total (*N* = 325)	Critical type (*N* = 103)	Severe type (*N* = 222)	*P* value
28-day mortality	42 (13%)	36 (35%)	6 (3%)	< 0.001
60-day mortality	55 (17%)	49 (48%)	6 (3%)	< 0.001

## Discussion

This retrospective case-control study identified for the first time the factors related to the risk of in-hospital death in Critical COVID-19 patients at high risk of death, and proposed the warning value for reference. Our results show that comorbidity, lymphopenia, higher SOFA score, and critical classification were associated with higher rates of in-hospital mortality. In particular, lymphocyte count < 0.8 × 109/L, SOFA score > 3, APACHE II score > 7, PaO2/FiO2 < 200 mmHg, IL-6 > 120 pg/ml, and CRP > 52 mg/L were associated with an increased risk of 28-day and 60-day mortality, and shorter survival time in critical patients who had a more severe inflammatory reaction and organ dysfunction.

In this group of Critical COVID-19 cases, the rate of in-hospital death was 17%. Those who died had more serious inflammatory indicators and organ damage than survivors, a finding consistent with a recent research report on the characteristics of critical COVID-19 patients who died [12]. In addition, nearly half of patients suffered a comorbidity, most commonly hypertension, followed by cardiovascular diseases and diabetes, very similar to characteristics of such patients in recent reports [13]. Recently, a meta-analysis of 6 studies revealed that hypertension, diabetes, chronic obstructive pulmonary disease, cardiovascular disease, and cerebrovascular disease are related independent risk factors for death in patients with COVID-19 [14]. Another meta-analysis showed that CRP, D-dimer, and LDH are elevated in patients with severe COVID-19 infection, while serum albumin levels are lower in severe illness compared with nonsevere COVID-19 infections, the cutoff for the parameters were 0.065 ng/ml for procalcitonin, 38.85 g/L for albumin,33.55 mg/L for CRP, 0.635 μ/L for D-dimer, and 263.5 U/L for LDH [15]. A study involving 1590 patients with COVID-19 showed that patients with two or more comorbidities had a significantly increased risk of poor prognosis compared to patients with no or only a single comorbidity [16]. Previously, immune disorders and prolonged inflammation were posited to the key factors for adverse outcomes in COVID-19 patients, and patients with circulatory and endocrine system diseases are more likely to have immune cell dysfunction and prolonged inflammation [17].

Previous studies on SARS-COV infection have shown that lymphopenia has been helpful in predicting the severity and clinical outcome of SARS-COV infection, which may be due to the fact that lymphocytes are directly infected and destroyed by SARS-COV [18]. Our study also found that, early in the disease course, non-survivors had significantly lower lymphocyte counts than did survivors, a result consistent with those of previous studies [19, 20]. Previously, pathological results of patients who died showed that a large number of lymphocytes and monocytes had infiltrated into the lungs of these patients [21], and we found, through Kaplan-Meier curve analysis, that patients with lymphocytes < 0.8 had an increased risk of death.

The SOFA score is currently the most commonly used method of assessing multi-organ dysfunction in the world [22], predicting mortality in sepsis. SOFA score was likewise an independent risk factor for 60-day survival after admission in COVID-19 patients in our study. Patients infected with SARS-COV-2 who presented with the diagnostic criteria of sepsis were more likely to suffer from severe lung injury and even multiple organ dysfunction, which provides supporting evidence for the hypothesis proposed by the latest research [23]. In this study, severe patients with infections and multiple organ dysfunction died mostly due to MODS at the end of the disease course. Considering that single organ failure is not the cause of death in COVID-19 patients, SOFA score can well predict the poor prognosis of patients, and the relevant Kaplan-Meier curve analysis of this study suggests that a SOFA score > 3 may indicate an increased risk of death for COVID-19 patients.

Based on Chinese Guidelines, patients are classified into severe and critical according to the severity of the disease. Patients classified as critical upon admission were at much greater risk of death, since such patients by definition have indications of a poor prognosis, including higher inflammatory indicators, more serious organ function damage, and higher SOFA score.

Inflammatory cytokines, such as IL-6, can cause the so-called “cytokine storm”, which may be a driver of acute lung injury and ARDS, and promote the progression of tissue damage to multiple organ failure [24]. In our subgroup analysis, IL-6 also was significantly increased in non-survivors, and the Kaplan-Meier curves showed that IL-6 > 120 pg/ml was associated with increased mortality. Our study also found a significantly different level of CRP between non-survivors and survivors, and previous studies have suggested that CRP level is an important indicator for diagnosing and evaluating severe pulmonary infectious diseases [25, 26]. In the early stage of COVID-19 infection, the CRP level can reflect the pulmonary lesions and the severity of the disease, and our study found that CRP > 52 mg/L indicated a poor prognosis. Our study showed that the PaO2/FiO2 level was lower in critical patients than in severe patients, and PaO2/FiO2 < 200 mmHg indicated a lower survival rate. SARS-COV-2 can attack the lung capillary endothelial cells, resulting in the exudation of a large amount of plasma components from the alveolar cavity, massive reduction in the number of lymphocytes and lymphocyte dysfunction, and infiltration of a large number of macrophages, further exacerbating lung injury [23]. Additionally, APACHE II score is one of the indicators used to evaluate the criticality of patients in the ICU [27], and in our study an APACHE II score > 7 indicated a lower survival rate.

In addition, different treatments could impact on mortality. Metformin has shown benefits in reducing the mortality rate from COVID-19 infection [28], while statin and tocilizumab use did not improve in-hospital outcomes [29, 30]. However, there are now two RCTs that report major mortality benefit with tocilizumab [31, 32]. In the latest April 14, 2021, Infectious Diseases Society of America Guidelines (IDSA) on the treatment and management of patients with COVID-19 revised Version4.2.0 of the guidelines suggest tocilizumab in addition to standard of care rather than standard of care alone [33]. In our other studies, glucocorticoid, thymosin α1 and immunoglobulin therapy were found to be associated with the outcome of critically ill patients with COVID-19 [34–37].

Our study has some limitations. Due to the retrospective study design and the small number of reported cases (critical cases and severe cases), not all laboratory tests were done in all patients, which may compromise the reliability of the statistical analysis. To verify the existing results, further research on an expanded sample size is compulsory.

## Conclusions

In all, comorbidity, lymphopenia, high SOFA score, and having a critical classification are risk factors for death in COVID-19 patients. Critical patients and patients with admission lymphocyte count < 0.8 × 109/L, SOFA score > 3, APACHE II score > 7, PaO2/FiO2 < 200 mmHg, IL-6 > 120 pg/ml, and CRP > 52 mg/L are more likely to have poor prognosis.

## Supplementary Information

**Additional file 1: Supplementary figure 1.** Survival curves of 60-day mortality in all patients with LYM>0.8 vs LYM≤0.8.

**Additional file 2: Supplementary figure 2.** Survival curves of 60-day mortality in all patients with SOFA>3.0 vs SOFA≤3.0.

**Additional file 3: Supplementary figure 3.** Survival curves of 60-day mortality in all patients with APACHEII>7.0 vs APACHEII≤7.0.

**Additional file 4: Supplementary figure 4.** Survival curves of 60-day mortality in all patients with Pa0_2_/FiO_2_>200 vs Pa0_2_/FiO_2_≤200.

**Additional file 5: Supplementary figure 5.** Survival curves of 60-day mortality in all patients with IL-6>120 vs IL-6≤120.

**Additional file 6: Supplementary figure 6.** Survival curves of 60-day mortality in all patients with CRP>52 vs CRP≤52.

**Additional file 7: Supplementary figure 7.** Survival curves of 60-day mortality in all patients with different clinical classifications.

**Additional file 8: Supplementary Table 1.** Clinical parameters in subgroups of LYM>0.8 vs LYM≤0.8.

**Additional file 9: Supplementary Table 2.** Clinical parameters in subgroups of SOFA>3.0 vs SOFA≤3.0.

**Additional file 10: Supplementary Table 3.** Clinical parameters in subgroups of APACHE II>7.0 vs APACHEII≤7.0.

**Additional file 11: Supplementary Table 4.** Clinical parameters in subgroups of Pa0_2_/FiO_2_>200 vs Pa0_2_/FiO_2_≤200.

**Additional file 12: Supplementary Table 5.** Clinical parameters in subgroups of IL-6>120vs IL-6≤120.

**Additional file 13: Supplementary Table 6.** Clinical parameters in subgroups of CRP>52 vs CRP≤52.

**Additional file 14: Supplementary Table 7.** Clinical parameters in subgroups of Crtical type vs Severe type.

## Data Availability

The data sets used and/or analyzed during the current study are available from the corresponding author on reasonable request.
