# Supplementary figures and images for: Risk factors for mortality in critically ill patients with COVID-19: a multicenter retrospective case-control study
Source: BMC Infect Dis. 2021 Jun 24;21:602. doi: 10.1186/s12879-021-06300-7 (PMC8223178; doi:10.1186/s12879-021-06300-7)

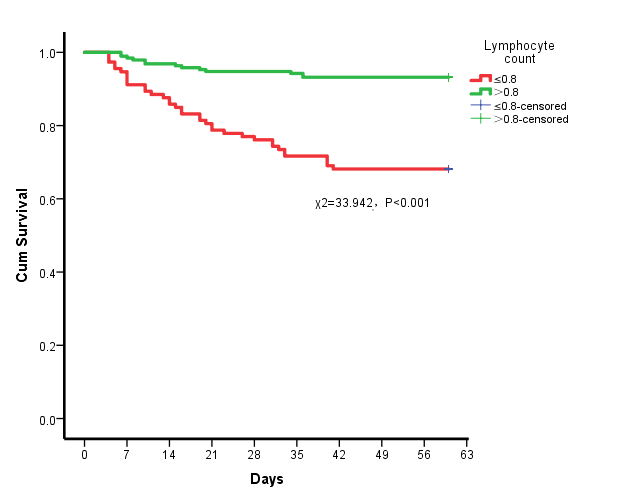


**Supplementary figure1:** **Survival curves of 60-day mortality in all patients with LYM＞0.8 vs LYM≤0.8**

Supplement: Supplementary file 1 — Additional file 1: Supplementary figure 1. Survival curves of 60-day mortality in all patients with LYM>0.8 vs LYM≤0.8. [file 12879_2021_6300_MOESM1_ESM.docx]

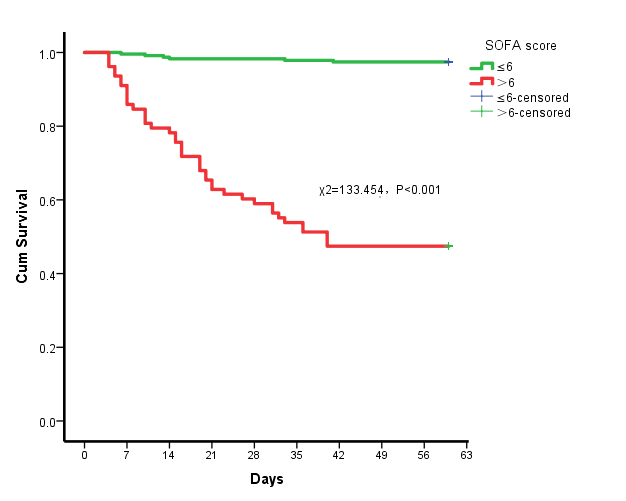


**Supplementary figure2:** **Survival curves of 60-day mortality in all patients with SOFA＞3.0 vs SOFA≤3.0**

Supplement: Supplementary file 2 — Additional file 2: Supplementary figure 2. Survival curves of 60-day mortality in all patients with SOFA>3.0 vs SOFA≤3.0. [file 12879_2021_6300_MOESM2_ESM.docx]

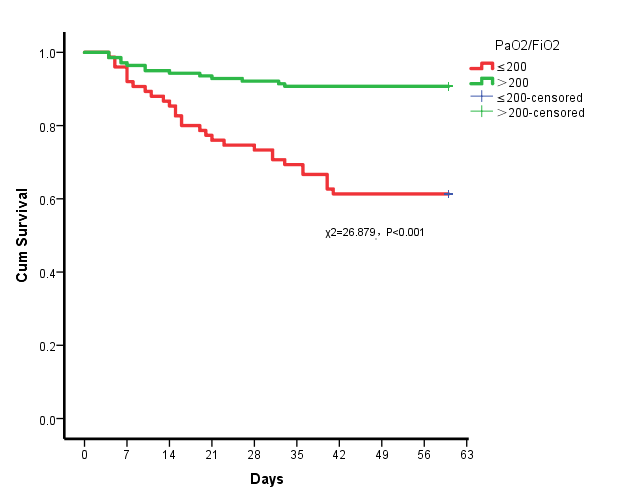


**Supplementary figure4: Survival curves of 60-day mortality in all patients with Pa02/FiO2＞200 vs Pa02/FiO2≤200**

Supplement: Supplementary file 4 — Additional file 4: Supplementary figure 4. Survival curves of 60-day mortality in all patients with Pa02/FiO2>200 vs Pa02/FiO2≤200. [file 12879_2021_6300_MOESM4_ESM.docx]

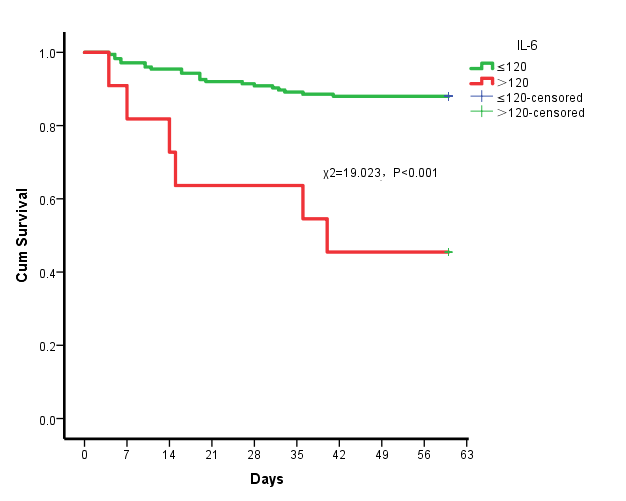


**Supplementary figure5: Survival curves of 60-day mortality in all patients with IL-6＞120 vs IL-6≤120**

Supplement: Supplementary file 5 — Additional file 5: Supplementary figure 5. Survival curves of 60-day mortality in all patients with IL-6>120 vs IL-6≤120. [file 12879_2021_6300_MOESM5_ESM.docx]

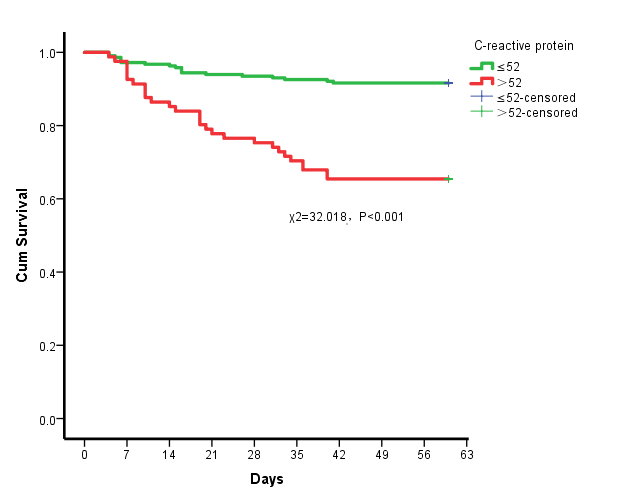


**Supplementary figure6: Survival curves of 60-day mortality in all patients with CRP＞52 vs CRP≤52**

Supplement: Supplementary file 6 — Additional file 6: Supplementary figure 6. Survival curves of 60-day mortality in all patients with CRP>52 vs CRP≤52. [file 12879_2021_6300_MOESM6_ESM.docx]
