# Supplementary material for: Risk factors for mortality in critically ill patients with COVID-19: a multicenter retrospective case-control study
Source: BMC Infect Dis. 2021 Jun 24;21:602. doi: 10.1186/s12879-021-06300-7 (PMC8223178; doi:10.1186/s12879-021-06300-7)
Supplement: Supplementary file 3 — Additional file 3: Supplementary figure 3. Survival curves of 60-day mortality in all patients with APACHEII>7.0 vs APACHEII≤7.0. [file 12879_2021_6300_MOESM3_ESM.docx]

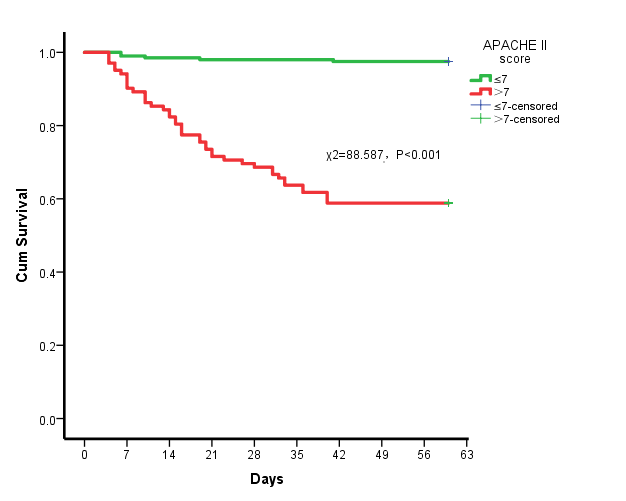


**Supplementary figure3:** **Survival curves of 60-day mortality in all patients with APACHEII＞7.0 vs APACHEII≤7.0**
