# Supplementary material for: Risk factors for mortality in critically ill patients with COVID-19: a multicenter retrospective case-control study
Source: BMC Infect Dis. 2021 Jun 24;21:602. doi: 10.1186/s12879-021-06300-7 (PMC8223178; doi:10.1186/s12879-021-06300-7)
Supplement: Supplementary file 8 — Additional file 8: Supplementary Table 1. Clinical parameters in subgroups of LYM>0.8 vs LYM≤0.8. [file 12879_2021_6300_MOESM8_ESM.docx]

| **Supplementary Table 1: Clinical parameters in subgroups of LYM＞0.8 vs LYM≤0.8** | | | |
| --- | --- | --- | --- |
| Variable | **LYM＞0.8**  **(N=191)** | **LYM≤0.8**  **(N=113)** | **P value** |
| **clinical parameters median(IQR)** | | | |
| WBC, (1×109/L) | 5.2(4.2-6.8) | 6.6(4.4-9.7) | 0.001 |
| NEU,(1×109/L) | 3.5(2.3-4.8) | 5.8(3.5-9.0) | <0.001 |
| MON,(1×109/L) | 0.5(0.4-0.7) | 0.3(0.2-0.5) | <0.001 |
| PLT,(1×109/L) | 188.0(148.0-241.0) | 163.0(124.0-215.5) | 0.008 |
| IL-6,(pg/ml) | 14.9(7.6-32.9) | 24.8(8.6-67.3) | 0.012 |
| PCT,(ng/ml) | 0.1(0-0.1) | 0.1(0.1-0.2) | <0.001 |
| CRP,(mg/L) | 16.4(6.0-42.7) | 45.6(19.4-82.3) | <0.001 |
| ALT, (U/L) | 23.0(15.6-35.0) | 28.0(19.0-49.0) | 0.001 |
| TBIL, (umol/L) | 10.1(7.1-14.2) | 13.0(9.6-18.8) | <0.001 |
| CREA, (µmol/L) | 65.1(52.1-80.0) | 66.0(52.5-83.5) | 0.608 |
| Lac, (mmol/L) | 1.4(1.1-1.9) | 1.9(1.4-2.6) | <0.001 |
| Pa0_2_/FiO_2_ | 250.0(206.0-291.4) | 179.0(118.0-261.1) | <0.001 |
| APACH II sore, median(IQR) | 5.0(3.0-8.0) | 8.0(5.0-10.0) | <0.001 |
| SOFA sore, median(IQR) | 2.0(1.0-3.0) | 3.0(2.0-5.0) | <0.001 |
| ^a^ Total course of disease：Time from illness onset to death or discharge, days ;APACHE II: Acute Physiology and Chronic Health Evaluation II score; SOFA: Sequential Organ Failure Assessment; WBC: White blood cell count; NEU: Neutrophil ; LYM :Lymphocyte count ; MON: Monocytes; PLT:Platelet count; HGB: Hemoglobin; FIB: Fibrinogen; IL-6: Interleutin-6; PCT: Procalcitonin; CRP: C-reactive protein; ALT: Alanine aminotransferase; TBIL: Total bilirubin; DBIL: Direct bilirubin; CREA: Creatine; Lac: lactic acid | | | |
