# Supplementary material for: Risk factors for mortality in critically ill patients with COVID-19: a multicenter retrospective case-control study
Source: BMC Infect Dis. 2021 Jun 24;21:602. doi: 10.1186/s12879-021-06300-7 (PMC8223178; doi:10.1186/s12879-021-06300-7)
Supplement: Supplementary file 9 — Additional file 9: Supplementary Table 2. Clinical parameters in subgroups of SOFA>3.0 vs SOFA≤3.0. [file 12879_2021_6300_MOESM9_ESM.docx]

| **Supplementary Table 2: Clinical parameters in subgroups of SOFA＞3.0 vs SOFA≤3.0** | | | |
| --- | --- | --- | --- |
| Variable | **SOFA＞3.0**  **(N=78)** | **SOFA≤3.0**  **(N=233)** | **P value** |
| **clinical parameters median(IQR)** |  |  |  |
| WBC, (1×109/L) | 8.7(5.1-11.6) | 5.2(4.0-6.7) | <0.001 |
| NEU,(1×109/L) | 7.2(4.3-10.1) | 5.5(2.4-5.1) | <0.001 |
| MON,(1×109/L) | 0.4(0.2-0.7) | 0.4(0.3-0.6) | 0.876 |
| LYM,(1×109/L) | 0.7(0.5-1.0) | 1.1(0.7-1.5) | <0.001 |
| PLT,(1×109/L) | 159.0(114.0-226.5) | 184.0(149.0-235.0) | 0.004 |
| IL-6,(pg/ml) | 36.8(10.5-74.1) | 16.2(6.9-33.0) | 0.001 |
| PCT,(ng/ml) | 0.2(0.1-0.3) | 0.1(0-0.1) | <0.001 |
| CRP,(mg/L) | 13.9(5.6-39.7) | 17.4(7.5-40.6) | <0.001 |
| ALT, (U/L) | 27.8(19.0-43.9) | 24.0(15.7-37.0) | 0.029 |
| TBIL, (umol/L) | 14.6(10.1-21.9) | 10.7(7.4-14.4) | <0.001 |
| CREA, (µmol/L) | 72.3(57.4-98.0) | 63.0(51.0-78.0) | <0.001 |
| Lac, (mmol/L) | 1.9(1.3-2.8) | 1.5(1.1-2.0) | 0.002 |
| Pa0_2_/FiO_2_ | 153.0(94.5-241.1) | 259.7(210.0-297.1) | <0.001 |
| APACH II sore, median(IQR) | 5.0(4.0-9.0) | 7.0(5.0-10.0) | <0.001 |
| APACHE II: Acute Physiology and Chronic Health Evaluation II score; SOFA: Sequential Organ Failure Assessment; WBC: White blood cell count; NEU: Neutrophil ; LYM :Lymphocyte count ; MON: Monocytes; PLT:Platelet count; HGB: Hemoglobin; FIB: Fibrinogen; IL-6: Interleutin-6; PCT: Procalcitonin; CRP: C-reactive protein; ALT: Alanine aminotransferase; TBIL: Total bilirubin; DBIL: Direct bilirubin; CREA: Creatine; Lac: lactic acid | | | |
