# Supplementary material for: Risk factors for mortality in critically ill patients with COVID-19: a multicenter retrospective case-control study
Source: BMC Infect Dis. 2021 Jun 24;21:602. doi: 10.1186/s12879-021-06300-7 (PMC8223178; doi:10.1186/s12879-021-06300-7)
Supplement: Supplementary file 10 — Additional file 10: Supplementary Table 3. Clinical parameters in subgroups of APACHE II>7.0 vs APACHEII≤7.0. [file 12879_2021_6300_MOESM10_ESM.docx]

| **Supplementary Table 3: Clinical parameters in subgroups of APACHE II＞7.0 vs APACHEII≤7.0** | | | |
| --- | --- | --- | --- |
| Variable | **APACHE II＞7.0**  **(N=102)** | **APACHEII≤7.0**  **(N=200)** | **P value** |
| **clinical parameters median(IQR)** |  |  |  |
| WBC, (1×109/L) | 7.4(5.0-10.7) | 5.2(4.0-6.8) | <0.001 |
| NEU,(1×109/L) | 5.9(3.5-8.9) | 3.4(2.3-5.1) | <0.001 |
| MON,(1×109/L) | 0.4(0.3-0.7) | 0.4(0.3-0.6) | 0.988 |
| LYM,(1×109/L) | 0.7(0.5-1.1) | 1.1(0.7-1.5) | <0.001 |
| PLT,(1×109/L) | 163.0(119.0-216.5) | 190.0(151.0-240.0) | 0.002 |
| IL-6,(pg/ml) | 40.1(15.4-88.0) | 13.0(6.6-24.8) | <0.001 |
| PCT,(ng/ml) | 0.1(0.1-0.2) | 0.1(0-0.1) | <0.001 |
| CRP,(mg/L) | 46.7(15.3-88.7) | 18.0(7.5-44.6) | <0.001 |
| ALT, (U/L) | 27.8(18.5-43.0) | 23.0(15.0-36.0) | 0.033 |
| TBIL, (umol/L) | 12.1(9.1-18.0) | 10.9(7.4-15.0) | 0.043 |
| CREA, (µmol/L) | 68.4(53.4-92.3) | 63.0(52.0-76.5) | 0.056 |
| Lac, (mmol/L) | 1.8(1.3-2.3) | 1.5(1.1-2.1) | 0.078 |
| Pa0_2_/FiO_2_ | 178.0(118.0-262.0) | 248.6(201.0-285.9) | <0.001 |
| SOFA sore, median(IQR) | 4.0(2.0-7.0) | 2.0(1.0-3.0) | <0.001 |
| APACHE II: Acute Physiology and Chronic Health Evaluation II score; SOFA: Sequential Organ Failure Assessment; WBC: White blood cell count; NEU: Neutrophil ; LYM :Lymphocyte count ; MON: Monocytes; PLT:Platelet count; HGB: Hemoglobin; FIB: Fibrinogen; IL-6: Interleutin-6; PCT: Procalcitonin; CRP: C-reactive protein; ALT: Alanine aminotransferase; TBIL: Total bilirubin; DBIL: Direct bilirubin; CREA: Creatine; Lac: lactic acid | | | |
