# Supplementary material for: Risk factors for mortality in critically ill patients with COVID-19: a multicenter retrospective case-control study
Source: BMC Infect Dis. 2021 Jun 24;21:602. doi: 10.1186/s12879-021-06300-7 (PMC8223178; doi:10.1186/s12879-021-06300-7)
Supplement: Supplementary file 11 — Additional file 11: Supplementary Table 4. Clinical parameters in subgroups of Pa02/FiO2>200 vs Pa02/FiO2≤200. [file 12879_2021_6300_MOESM11_ESM.docx]

| **Supplementary Table 4: Clinical parameters in subgroups of Pa0_2_/FiO_2_＞200 vs Pa0_2_/FiO_2_≤200** | | | |
| --- | --- | --- | --- |
| Variable | Pa0_2_/FiO_2_＞200  (N=140) | Pa0_2_/FiO_2_≤200  (N=75) | P value |
| **clinical parameters median(IQR)** |  |  |  |
| WBC, (1×109/L) | 5.4(4.3-7.0) | 7.6(5.1-10.5) | <0.001 |
| NEU,(1×109/L) | 3.7(2.6-5.3) | 6.0(3.7-9.1) | <0.001 |
| MON,(1×109/L) | 0.4(0.3-0.6) | 0.4(0.2-0.6) | 0.154 |
| LYM,(1×109/L) | 1.1(0.8-1.5) | 0.7(0.5-0.9) | <0.001 |
| PLT,(1×109/L) | 176.0(138.8-231.0) | 177.5(147.3-239.5) | 0.934 |
| IL-6,(pg/ml) | 13.6(5.0-37.7) | 48.1(16.4-83.0) | <0.001 |
| PCT,(ng/ml) | 0.1(0-0.2) | 0.1(0.1-0.3) | <0.001 |
| CRP,(mg/L) | 25.1(8.6-46.4) | 52.4(24.3-109.0) | <0.001 |
| ALT, (U/L) | 24.0(16.0-36.7) | 31.7(19.7-50.3) | 0.006 |
| TBIL, (umol/L) | 12.0(7.9-16.4) | 13.0(9.2-19.2) | 0.164 |
| CREA, (µmol/L) | 66.0(52.6-81.6) | 67.0(55.0-90.5) | 0.492 |
| Lac, (mmol/L) | 1.5(1.1-2.1) | 1.8(1.3-2.5) | 0.009 |
| APACHEII sore, median(IQR) | 6.0(3.3-8.0) | 9.0(6.0-12.0) | <0.001 |
| SOFA sore, median(IQR) | 2.0(2.0-3.0) | 4.0(3.0-6.0) | <0.001 |
| APACHE II: Acute Physiology and Chronic Health Evaluation II score; SOFA: Sequential Organ Failure Assessment; WBC: White blood cell count; NEU: Neutrophil ; LYM :Lymphocyte count ; MON: Monocytes; PLT:Platelet count; HGB: Hemoglobin; FIB: Fibrinogen; IL-6: Interleutin-6; PCT: Procalcitonin; CRP: C-reactive protein; ALT: Alanine aminotransferase; TBIL: Total bilirubin; DBIL: Direct bilirubin; CREA: Creatine; Lac: lactic acid | | | |
