# Supplementary material for: Risk factors for mortality in critically ill patients with COVID-19: a multicenter retrospective case-control study
Source: BMC Infect Dis. 2021 Jun 24;21:602. doi: 10.1186/s12879-021-06300-7 (PMC8223178; doi:10.1186/s12879-021-06300-7)
Supplement: Supplementary file 12 — Additional file 12: Supplementary Table 5. Clinical parameters in subgroups of IL-6>120vs IL-6≤120. [file 12879_2021_6300_MOESM12_ESM.docx]

| **Supplementary Table 5: Clinical parameters in subgroups of IL-6＞120vs IL-6≤120** | | | |
| --- | --- | --- | --- |
| Variable | IL-6＞120  **(N=11)** | IL-6≤120  **(N=175)** | **P value** |
| **clinical parameters median(IQR)** |  |  |  |
| WBC, (1×109/L) | 6.1(5.0-10.6) | 5.3(4.0-7.9) | 0.239 |
| NEU,(1×109/L) | 4.0(2.7-8.8) | 3.7(2.4-6.0) | 0.522 |
| LYM,(1×109/L) | 0.9(0.7-1.3) | 0.9(0.6-1.4) | 0.710 |
| MON,(1×109/L) | 0.5(0.2-0.8) | 0.4(0.3-0.6) | 0.633 |
| PLT,(1×109/L) | 167.0(80.0-195.0) | 175.0(144.8-233.3) | 0.178 |
| PCT,(ng/ml) | 0.2(0.1-0.2) | 0.1(0-0.1) | 0.001 |
| CRP,(mg/L) | 71.6(38.8-95.7) | 22.4(8.7-60.2) | 0.018 |
| ALT, (U/L) | 30.0(18.0-51.0) | 25.5(16.7-36.9) | 0.704 |
| DBIL, (umol/L) | 4.7(3.0-7.1) | 3.5(2.1-4.9) | 0.079 |
| CREA, (µmol/L) | 80.0(75.0-106.0) | 65.5(54.0-81.2) | 0.014 |
| Lac, (mmol/L) | 1.4(1.0-1.6) | 1.5(1.1-2.1) | 0.334 |
| Pa0_2_/FiO_2_ | 148.6(100.2-254.3) | 244.0(168-283.1) | 0.065 |
| APACH II sore, median(IQR) | 11.0(10.0-28.0) | 6.0(4.0-8.5) | <0.001 |
| SOFA sore, median(IQR) | 4.0(2.0-12.0) | 2.0(2.0-3.0) | 0.030 |
| APACHE II: Acute Physiology and Chronic Health Evaluation II score; SOFA: Sequential Organ Failure Assessment; WBC: White blood cell count; NEU: Neutrophil ; LYM :Lymphocyte count ; MON: Monocytes; PLT:Platelet count; HGB: Hemoglobin; FIB: Fibrinogen; IL-6: Interleutin-6; PCT: Procalcitonin; CRP: C-reactive protein; ALT: Alanine aminotransferase; TBIL: Total bilirubin; DBIL: Direct bilirubin; CREA: Creatine; Lac: lactic acid | | | |
