# Supplementary material for: Risk factors for mortality in critically ill patients with COVID-19: a multicenter retrospective case-control study
Source: BMC Infect Dis. 2021 Jun 24;21:602. doi: 10.1186/s12879-021-06300-7 (PMC8223178; doi:10.1186/s12879-021-06300-7)
Supplement: Supplementary file 13 — Additional file 13: Supplementary Table 6. Clinical parameters in subgroups of CRP>52 vs CRP≤52. [file 12879_2021_6300_MOESM13_ESM.docx]

| **Supplementary Table 6: Clinical parameters in subgroups of CRP＞52 vs CRP≤52** | | | |
| --- | --- | --- | --- |
| Variable | CRP＞52  **(N=81)** | CRP≤52  **(N=215)** | **P value** |
| **clinical parameters median(IQR)** |  |  |  |
| WBC, (1×109/L) | 6.8(5.0-10.6) | 5.3(4.0-7.1) | <0.001 |
| NEU,(1×109/L) | 5.4(3.6-9.1) | 3.5(2.3-5.4) | <0.001 |
| LYM,(1×109/L) | 0.7(0.5-1.0) | 1.1(0.7-1.6) | <0.001 |
| MON,(1×109/L) | 0.4(0.2-0.7) | 0.4(0.3-0.6) | 0.311 |
| PLT,(1×109/L) | 171.0(126.0-215.0) | 181.0(145.0-237.8) | 0.070 |
| IL-6,(pg/ml) | 48.1(21.6-90.3) | 12.4(6.5-27.8) | <0.001 |
| PCT,(ng/ml) | 0.2(0.1-0.3) | 0.1(0-0.1) | <0.001 |
| ALT, (U/L) | 30.0(19.5-43.4) | 23.0(15.6-36.0) | 0.011 |
| TBIL, (umol/L) | 13.8(10.1-18.7) | 10.5(7.4-14.5) | <0.001 |
| CREA, (µmol/L) | 69.0(54.8-87.3) | 63.3(52.0-80.0) | 0.088 |
| Lac, (mmol/L) | 1.7(1.3-2.3) | 1.5(1.1-2.2) | 0.241 |
| Pa0_2_/FiO_2_ | 176.5(130.0-250.0) | 248.8(201.5-290.7) | <0.001 |
| APACH II sore, median(IQR) | 8.0(6.0-11.0) | 5.0(3.0-8.0) | <0.001 |
| SOFA sore, median(IQR) | 4.0(2.0-6.0) | 2.0(1.0-3.0) | <0.001 |
| APACHE II: Acute Physiology and Chronic Health Evaluation II score; SOFA: Sequential Organ Failure Assessment; WBC: White blood cell count; NEU: Neutrophil ; LYM :Lymphocyte count ; MON: Monocytes; PLT:Platelet count; HGB: Hemoglobin; FIB: Fibrinogen; IL-6: Interleutin-6; PCT: Procalcitonin; CRP: C-reactive protein; ALT: Alanine aminotransferase; TBIL: Total bilirubin; DBIL: Direct bilirubin; CREA: Creatine; Lac: lactic acid | | | |
