# Supplementary material for: Risk factors for mortality in critically ill patients with COVID-19: a multicenter retrospective case-control study
Source: BMC Infect Dis. 2021 Jun 24;21:602. doi: 10.1186/s12879-021-06300-7 (PMC8223178; doi:10.1186/s12879-021-06300-7)
Supplement: Supplementary file 14 — Additional file 14: Supplementary Table 7. Clinical parameters in subgroups of Crtical type vs Severe type. [file 12879_2021_6300_MOESM14_ESM.docx]

| **Supplementary Table 7: Clinical parameters in subgroups of Crtical type vs Severe type** | | | |
| --- | --- | --- | --- |
| Variable | Critical type  **(N=103)** | Severe type  **(N=222)** | **P value** |
| **clinical parameters median(IQR)** |  |  |  |
| WBC, (1×109/L) | 7.8(5.6-11.0) | 5.0(4.0-6.6) | <0.001 |
| NEU,(1×109/L) | 6.5(4.2-9.1) | 3.2(2.3-4.9) | <0.001 |
| LYM,(1×109/L) | 0.7(0.5-1.0) | 1.1(0.7-1.6) | <0.001 |
| MON,(1×109/L) | 0.4(0.3-0.6) | 0.4(0.3-0.6) | 0.843 |
| PLT,(1×109/L) | 173.0(119.0-215.0) | 183.0(146.0-236.5) | 0.047 |
| IL-6,(pg/ml) | 30.3(9.6-75.6) | 16.0(7.6-33.0) | 0.003 |
| PCT,(ng/ml) | 0.1(0.1-0.2) | 0.1(0-0.1) | <0.001 |
| CRP,(mg/L) | 47.3(24.9-92.4) | 15.5(6.3-42.2) | <0.001 |
| ALT, (U/L) | 25.0(18.0-39.0) | 24.0(16.0-37.1) | 0.462 |
| TBIL, (umol/L) | 13.8(10.2-20.6) | 10.0(7.3-14.5) | <0.001 |
| CREA, (µmol/L) | 72.4(56.6-94.6) | 62.5(51.2-76.3) | <0.001 |
| Lac, (mmol/L) | 2.1(1.5-2.7) | 1.4(1.1-1.8) | <0.001 |
| Pa0_2_/FiO_2_ | 195.4(130.5-289.0) | 247.7(204.6-283.1) | 0.003 |
| APACH II sore, median(IQR) | 8.0(6.0-18.0) | 5.0(3.0-7.0) | <0.001 |
| SOFA sore, median(IQR) | 4.0(3.0-7.3) | 2.0(1.0-2.0) | <0.001 |
| APACHE II: Acute Physiology and Chronic Health Evaluation II score; SOFA: Sequential Organ Failure Assessment; WBC: White blood cell count; NEU: Neutrophil ; LYM :Lymphocyte count ; MON: Monocytes; PLT:Platelet count; HGB: Hemoglobin; FIB: Fibrinogen; IL-6: Interleutin-6; PCT: Procalcitonin; CRP: C-reactive protein; ALT: Alanine aminotransferase; TBIL: Total bilirubin; DBIL: Direct bilirubin; CREA: Creatine; Lac: lactic acid | | | |
